# Supplementary material for: Parental Hesitancy towards the Established Childhood Vaccination Programmes in the COVID-19 Era: Assessing the Drivers of a Challenging Public Health Concern
Source: Vaccines (Basel). 2022 May 20;10(5):814. doi: 10.3390/vaccines10050814 (PMC9144671; doi:10.3390/vaccines10050814)
Supplement: Supplementary file 1 [file vaccines-10-00814-s001.zip › Table S1.pdf]

Table S1: Socio-demographic, lifestyle and health-related characteristics of participants and COVID-19 related variables, according to sex.

	Men N=380 (34.7%)	Women N=715 (65.3%)	p-value
**Childhood vaccine hesitancy**	34 (8.9)	64 (9.0)	>0.99
**Age (years), mean (SD)**	51.53 (9.23)	49.57 (9.37)	0.001
**BMI (kg/m²), mean (SD)** [1]	27.75 (4.30)	25.46 (4.66)	1.6×10^{-14}
**Total METs [kcal/(kg×h)], mean (SD)** [2]	25.74 (36.81)	19.03 (24.94)	0.001
**Marital status, N (%)**			
Unmarried/divorced/widowed	41 (10.9)	114 (16.1)	
Married/in cohabitation agreement	335 (89.1)	593 (83.9)	0.020
**Education, N (%)**			
Up to high school	100 (26.4)	178 (25.0)	
University degree	153 (40.4)	305 (42.9)	
Master's degree or higher	126 (33.2)	228 (32.1)	0.720
**Profession, N (%)**			
Executive/scientist/artist/office	238 (66.5)	531 (81.4)	
Providing services/seller	29 (8.1)	70 (10.7)	
Manual labour	91 (25.4)	51 (7.8)	1.3×10^{-13}
**Income (Euros/month), N (%)**			
≤900	70 (19.8)	200 (31.4)	
901-1100	52 (14.7)	125 (19.7)	
1101-1400	97 (27.5)	196 (30.8)	
>1400	134 (38.0)	115 (18.1)	5.5×10^{-11}
**Smoking status, N (%)**			
Non-smokers	122 (32.2)	292 (41.0)	
Former smokers	120 (31.7)	136 (19.1)	
Current smokers	137 (36.1)	284 (39.9)	1.2×10^{-05}
**Alcohol consumption, N (%)**			
Never	24 (6.3)	148 (20.8)	
Less than once/month	94 (24.7)	224 (31.5)	
1-3 times/month	103 (27.1)	150 (21.1)	
1-2 times/week	95 (25.0)	139 (19.5)	
3 or more times/week	64 (16.8)	51 (7.2)	1.4×10^{-13}
**Weight change during last 6 months, N (%)**			
No change	224 (58.9)	350 (49.4)	
Lost weight	67 (17.6)	129 (18.2)	
Gained weight	89 (23.4)	229 (32.3)	0.004
**Health status, N (%)**			
Moderate/bad/very bad	44 (11.6)	108 (15.1)	
Good	213 (56.3)	360 (50.5)	
Very good	121 (32.0)	245 (34.4)	0.122
**Restriction of activities during last year due to a health issue, N (%)**			
None	243 (64.1)	379 (53.9)	
A little/moderately	94 (24.8)	197 (28.0)	
Much/very much	42 (11.1)	127 (18.1)	0.001
**Stress during last 2 weeks, N (%)**			
No days	98 (25.8)	94 (13.1)	
Some days	199 (52.4)	408 (57.1)	
More than half days	42 (11.1)	102 (14.3)	
Almost every day	41 (10.8)	111 (15.5)	1.7×10^{-06}
**Depression during last 2 weeks, N (%)**			
No days	172 (45.3)	202 (28.3)	
Some days	147 (38.7)	369 (51.6)	
More than half days	39 (10.3)	83 (11.6)	
Almost every day	22 (5.8)	61 (8.5)	3.8×10^{-07}
**Chronic disease, N (%)**	133 (35.8)	259 (36.8)	0.748
**Diabetes diagnosis, N (%)**	29 (7.7)	50 (7.0)	0.689
**High cholesterol diagnosis, N (%)**	155 (41.0)	262 (36.8)	0.174

**High blood pressure diagnosis, N (%)**	104 (27.6)	133 (18.6)	0.001
**Area of residence, N (%)**			
Attica	70 (18.5)	140 (19.7)	
Peloponnese	20 (5.3)	28 (3.9)	
Islands (Aegean and Ionian islands, Crete)	13 (3.4)	26 (3.7)	
Thessaly and Central Greece	14 (3.7)	34 (4.8)	
Epirus	215 (56.9)	397 (55.9)	
Macedonia and Thrace	46 (12.2)	85 (12.0)	0.857

Questions about COVID-19

**Coronavirus existence, N (%)**			
Definitely not/probably not/do not know	17 (4.5)	25 (3.5)	
Probably yes	45 (11.8)	90 (12.6)	
Definitely yes	318 (83.7)	600 (83.9)	0.694
**Knowledge about COVID-19, N (%)**			
Poor knowledge	109 (28.7)	210 (29.4)	
Moderate knowledge	39 (10.3)	106 (14.8)	
Good knowledge	232 (61.1)	399 (55.8)	0.077
**Following COVID-19 measures of personal protection, N (%)** [3,4]			
Never/rarely/sometimes	25 (6.6)	38 (5.3)	
Frequently	85 (22.4)	77 (10.8)	
Almost always/always	269 (71.0)	599 (83.9)	6.3×10^{-07}
**Following COVID-19 measures of public protection, N (%)** [3,5]			
Never/rarely/sometimes	15 (4.0)	33 (4.6)	
Frequently	59 (15.6)	54 (7.6)	
Almost always/always	303 (80.4)	624 (87.8)	1.8×10^{-04}
**Trust to health authorities for minimizing the spread of coronavirus, N (%)** [3]			
No trust	63 (16.8)	111 (16.2)	
Little trust	75 (19.9)	126 (18.4)	
Some trust	139 (37.0)	316 (46.1)	
Absolute trust	99 (26.3)	133 (19.4)	0.015
**Trust to official information for the new pandemic, N (%)** [3]			
No trust	131 (34.7)	206 (29.5)	
Little trust	73 (19.4)	161 (23.1)	
Some/absolute trust	173 (45.9)	331 (47.4)	0.149
**Trust to Government for minimizing the spread of coronavirus, N (%)** [3]			
No trust	136 (36.7)	221 (32.5)	
Little trust	67 (18.1)	134 (19.7)	
Some trust	98 (26.4)	241 (35.5)	
Absolute trust	70 (18.9)	83 (12.2)	0.002
**Seasonal flu vaccination this year, N (%)** [3]			
No	157 (44.0)	299 (45.4)	
Yes	200 (56.0)	360 (54.6)	0.670
**Seasonal flu vaccination last year, N (%)** [3]			
No	233 (61.5)	441 (61.8)	
Yes	146 (38.5)	273 (38.2)	0.926
**Capability to protect against coronavirus, N (%)** [3]			
No/little capability	26 (7.0)	38 (5.5)	
Moderate capability	226 (60.8)	445 (64.0)	
Absolute capability	120 (32.3)	212 (30.5)	0.452
**COVID-19 symptoms during last months, N (%)** [3,6]			
No	371 (97.6)	668 (94.0)	
Yes	9 (2.4)	43 (6.0)	0.007
**COVID-19 tested, N (%)** [3]			
No	247 (65.3)	462 (64.9)	
Yes	131 (34.7)	250 (35.1)	0.880
**Family member with COVID-19 diagnosis, N (%)** [3]			
No	362 (95.3)	666 (93.4)	
Yes	18 (4.7)	47 (6.6)	0.217

**Willingness to vaccinate against coronavirus, N (%)**			
No	96 (25.3)	219 (30.6)	
Yes	226 (59.5)	323 (45.2)	1.7×10^{-05}
Don’t know/Don’t answer	58 (15.3)	173 (24.2)	
**Willingness to vaccinate their children against coronavirus, N (%)**			
No	121 (31.8)	274 (38.3)	
Yes	181 (47.6)	250 (35.0)	2.2×10^{-04}
Don’t know/Don’t answer	78 (20.5)	191 (26.7)	

Abbreviations: BMI, Body mass index; COVID-19, coronavirus disease 2019; SD, Standard deviation.

[1] Men: N=365; women: N=693.

[2] Men: N=316; women: N=612.

[3] The responses “Do not know/Do not answer” were < 10% of the total number of responses and were not taken into account

[4] COVID-19 measures of personal protection: mask use, frequent hand washing, keeping distance

[5] COVID-19 measures of public protection: mask use, covering the nose / mouth when coughing or sneezing, avoiding large concentrations, staying home or informing the authorities when not feeling well

[6] Fever, persistent cough, breathing difficulty, loss of smell / taste
