# Supplementary material for: Parental Hesitancy towards the Established Childhood Vaccination Programmes in the COVID-19 Era: Assessing the Drivers of a Challenging Public Health Concern
Source: Vaccines (Basel). 2022 May 20;10(5):814. doi: 10.3390/vaccines10050814 (PMC9144671; doi:10.3390/vaccines10050814)
Supplement: Supplementary file 1 [file vaccines-10-00814-s001.zip › Table S2.pdf]

Table S2: Odds ratios (OR) and 95% confidence intervals (CI) for the association between considerations about COVID-19 and childhood vaccine hesitancy, using post code as a cluster variable.

	**Minimally adjusted model** [1]		**Maximally adjusted model** [2]	
	**OR (95% CI)**	**p-value**	**OR (95% CI)**	**p-value**
**Coronavirus existence**				
Definitely not/probably not/do not know	Ref		Ref	
Probably yes	0.21 (0.11, 0.42)		0.20 (0.06, 0.64)	
Definitely yes	0.05 (0.04, 0.07)	1.3×10^{-115}	0.05 (0.03, 0.09)	4.0×10^{-59}
**Knowledge about COVID-19**				
Poor knowledge	Ref		Ref	
Moderate knowledge	0.64 (0.40, 1.02)		0.48 (0.32, 0.72)	
Good knowledge	0.45 (0.34, 0.59)	7.1×10^{-09}	0.49 (0.27, 0.89)	1.8×10^{-04}
**Following COVID-19 measures of personal protection** [3, 4]				
Never/rarely/sometimes	Ref		Ref	
Frequently	0.22 (0.09, 0.55)		0.33 (0.08, 1.44)	
Almost always/always	0.06 (0.04, 0.08)	1.4×10^{-92}	0.09 (0.05, 0.15)	5.8×10^{-108}
**Following COVID-19 measures of public protection** [3, 5]				
Never/rarely/sometimes	Ref		Ref	
Frequently	0.22 (0.10, 0.49)		0.23 (0.06, 0.94)	
Almost always/always	0.06 (0.03, 0.09)	3.4×10^{-41}	0.06 (0.02, 0.16)	2.5×10^{-29}
**Trust to health authorities for minimizing the spread of coronavirus** [3]				
No trust	Ref		Ref	
Little trust	0.28 (0.10, 0.80)		0.26 (0.11, 0.62)	
Some trust	0.14 (0.08, 0.24)		0.12 (0.05, 0.27)	
Absolute trust	0.05 (0.02, 0.15)	3.8×10^{-17}	0.04 (0.00, 0.41)	1.2×10^{-09}
**Trust to official information for the new pandemic** [3]				
No trust	Ref		Ref	
Little trust	0.31 (0.18, 0.51)		0.48 (0.29, 0.79)	
Some/absolute trust	0.11 (0.04, 0.29)	2.5×10^{-07}	0.08 (0.02, 0.34)	9.8×10^{-05}
**Trust to Government for minimizing the spread of coronavirus** [3]				
No trust	Ref		Ref	

Little trust	0.32 (0.11, 0.92)		0.47 (0.13, 1.65)	
Some trust	0.21 (0.08, 0.59)		0.18 (0.03, 1.03)	
Absolute trust	0.08 (0.02, 0.32)	6.6×10^{-12}	0.05 (0.01, 0.18)	8.1×10^{-16}
**Seasonal flu vaccination this year** [3]				
No	Ref		Ref	
Yes	0.08 (0.04, 0.19)	4.9×10^{-09}	0.08 (0.01, 0.39)	0.002
**Seasonal flu vaccination last year** [3]				
No	Ref		Ref	
Yes	0.18 (0.09, 0.37)	3.7×10^{-06}	0.18 (0.05, 0.67)	0.011
**Capability to protect against coronavirus** [3]				
No/little capability	Ref		Ref	
Moderate capability	1.39 (0.76, 2.54)		1.21 (0.91, 1.60)	
Absolute capability	2.84 (0.93, 8.69)	0.185 *	1.79 (0.65, 4.97)	0.300 *
**COVID-19 symptoms during last months** [3, 6]				
No	Ref		Ref	
Yes	1.84 (1.06, 3.19)	0.029	0.82 (0.09, 7.90)	0.867 *
**COVID-19 tested** [3]				
No	Ref		Ref	
Yes	0.75 (0.47, 1.19)	0.218 *	1.12 (0.61, 2.04)	0.721 *
**Family member with COVID-19 diagnosis** [3]				
No	Ref		Ref	
Yes	0.69 (0.27, 1.75)	0.432 *	0.76 (0.09, 6.76)	0.806 *
**Willingness to vaccinate against coronavirus** [3]				
No	Ref			
Yes	0.01 (0.00, 0.02)	2.5×10^{-11}	‡	
**Willingness to vaccinate their children against coronavirus** [3]				
No	Ref		Ref	
Yes	0.02 (0.01, 0.03)	1.6×10^{-48}	0.02 (0.00, 0.10)	1.4×10^{-05}

Abbreviations: COVID-19, coronavirus disease 2019; Ref, reference category

[1] Model adjusted for age, sex, education, and income

[2] Model adjusted for age, sex, education, income, depression, physical activity measured using total metabolic equivalents, profession, health status, smoking status and body mass index

[3] The responses “Do not know/Do not answer” were not taken into account

[4] COVID-19 measures of personal protection: mask use, frequent hand washing, keeping distance

[5] COVID-19 measures of public protection: mask use, covering the nose/mouth when coughing or sneezing, avoiding large concentrations, staying home or informing the authorities when not feeling well

[6] Fever, persistent cough, breathing difficulty, loss of smell/taste

* p-value not statistically significant after FDR correction for multiple comparisons.

‡ Model could not run because the variable predicted failures perfectly
