# Supplementary material for: Parental Hesitancy towards the Established Childhood Vaccination Programmes in the COVID-19 Era: Assessing the Drivers of a Challenging Public Health Concern
Source: Vaccines (Basel). 2022 May 20;10(5):814. doi: 10.3390/vaccines10050814 (PMC9144671; doi:10.3390/vaccines10050814)
Supplement: Supplementary file 1 [file vaccines-10-00814-s001.zip › Table S3.pdf]

Table S3: Betas and 95% confidence intervals (CI) for the association between COVID-19 related variables and change in the childhood vaccine hesitancy score for the 156 participants that responded to the second questionnaire, using univariable models.

	N (%)	beta (95% CI)	p-value
**Coronavirus existence**			
Definitely not/probably not/do not know/probably yes	17 (10.9)	Ref	
Definitely yes	139 (89.1)	4.76 (-1.00, 10.51)	0.11
**Knowledge about COVID-19**			
Poor knowledge	33 (21.2)	Ref	
Moderate knowledge	15 (9.6)	-2.28 (-9.33, 4.76)	
Absolute knowledge	108 (69.2)	-1.28 (-5.78, 3.22)	0.78
**Following COVID-19 measures of personal protection** [1]			
Never/rarely/sometimes/frequently	30 (19.2)	Ref	
Almost always/always	126 (80.8)	0.09 (-4.50, 4.67)	0.97
**Following COVID-19 measures of public protection** [2]			
Never/rarely/sometimes/frequently	20 (12.8)	Ref	
Almost always/always	136 (87.2)	-0.37 (-5.78, 5.04)	0.89
**Trust to health authorities for minimizing the spread of coronavirus** [3]			
No trust	10 (6.5)	Ref	
Little trust	18 (11.6)	2.89 (-5.98, 11.75)	
Some trust	85 (54.8)	-0.33 (-7.85, 7.18)	
Absolute trust	42 (27.1)	-1.95 (-9.86, 5.96)	0.51
**Trust to official information for the new pandemic**			
No trust	29 (18.6)	Ref	
Little trust	31 (19.9)	-1.68 (-7.51, 4.14)	
Some/absolute trust	96 (61.5)	-3.11 (-7.88, 1.67)	0.42
**Trust to Government for minimizing the spread of coronavirus** [3]			
No trust	26 (17.3)	Ref	
Little trust	31 (20.7)	-4.29 (-10.29, 1.71)	
Some trust	66 (44)	-5.07 (-10.29, 0.15)	
Absolute trust	27 (18)	-4.62 (-10.82, 1.58)	0.28
**Seasonal flu vaccination this year** [3]			

No	70 (48.6)	Ref	
Yes	74 (51.4)	1.36 (-2.42, 5.15)	0.48
**Seasonal flu vaccination last year** [3]			
No	111 (71.6)	Ref	
Yes	44 (28.4)	1.92 (-2.10, 5.95)	0.35
**Capability to protect against coronavirus**			
No/little capability	12 (7.7)	Ref	
Moderate capability	100 (64.1)	-3.47 (-10.28, 3.35)	
Absolute capability	44 (28.2)	0.83 (-6.43, 8.10)	0.09
**COVID-19 symptoms during last months** [3, 4]			
No	147 (95.4)	Ref	
Yes	7 (4.6)	5.81 (-2.89, 14.50)	0.19
**COVID-19 tested**			
No	93 (59.6)	Ref	
Yes	63 (40.4)	0.21 (-3.48, 3.89)	0.91
**Family member with COVID-19 diagnosis**			
No	146 (93.6)	Ref	
Yes	10 (6.4)	4.08 (-3.27, 11.44)	0.27
**Willingness to vaccinate against coronavirus** [3]			
No	26 (21.3)	Ref	
Yes	96 (78.7)	0.82 (-3.96, 5.59)	0.74
**Willingness to vaccinate their children against coronavirus** [3]			
No	40 (35.1)	Ref	
Yes	74 (64.9)	0.77 (-3.71, 5.25)	0.73

Abbreviations: COVID-19, coronavirus disease 2019; Ref, reference category.

[1] COVID-19 measures of personal protection: mask use, frequent hand washing, keeping distance

[2] COVID-19 measures of public protection: mask use, covering the nose/mouth when coughing or sneezing, avoiding large concentrations, staying home or informing the authorities when not feeling well

[3] The responses “Do not know/Do not answer” were not taken into account

[4] Fever, persistent cough, breathing difficulty, loss of smell/taste
