# Supplementary material for: Parental Hesitancy towards the Established Childhood Vaccination Programmes in the COVID-19 Era: Assessing the Drivers of a Challenging Public Health Concern
Source: Vaccines (Basel). 2022 May 20;10(5):814. doi: 10.3390/vaccines10050814 (PMC9144671; doi:10.3390/vaccines10050814)
Supplement: Supplementary file 1 [file vaccines-10-00814-s001.zip › Text S1.pdf]

**Text S1**

Description of COVID-19 related variables

Participants were asked to report if they have had symptoms of COVID-19, whether they have been diagnosed or tested against it and if a family member had been diagnosed with it (3 questions); their personal beliefs regarding coronavirus existence and the level of their knowledge regarding COVID-19 (2 questions); the use of personal or community public health mitigation measures (i.e. mask use, frequent hand washing, keeping distance, covering the nose / mouth when coughing or sneezing, avoiding large concentrations, staying home or informing the authorities when not feeling well (2 questions); the level of their trust to the government, health authorities and official information sources regarding the management of the pandemic (3 questions); their personal capability to protect themselves from COVID-19 (1 question); whether they had been vaccinated against seasonal flu the current or the previous year (2 questions); and how willing they were to vaccinate themselves or their children against SARS-CoV-2 (2 questions).

Description of adjusting variables in minimally and maximally adjusted models

The adjusting variables in the minimally adjusted model were age (as a continuous variable), sex, education (up to high school; university degree; Master's degree or higher), and monthly income (≤900; 901-1100; 1101-1400; >1400 Euros).

In the maximally adjusted model further adjustment was performed for presence of depressive symptoms during the last 2 weeks (no days; some days; more than half days; almost every day), recreational physical activity (walking, moderate, and vigorous measured in times/week and hours/day and converted to total metabolic equivalents – MET; a MET score of 3 for walking, 6 for moderate- and 9.0 for vigorous-intensity physical activity was used [1]), profession (executive/scientist/artist/office; providing services/seller; manual labour), health status (moderate/bad/very bad; good; very good), smoking status (non-smokers; former smokers; current smokers) and continuous body mass index (BMI).

1. Ainsworth, B.E., et al., Compendium of physical activities: classification of energy costs of human physical activities. *Med Sci Sports Exerc*, 1993. **25**(1): p. 71-80.
